# Erythema at the bacillus Calmette-Guerin scar after influenza vaccination

**DOI:** 10.1590/0037-8682-0390-2019

**Published:** 2019-12-20

**Authors:** Yanin Chavarri-Guerra, Enrique Soto-Perez-de-Celis

**Affiliations:** 1 Instituto Nacional de Ciencias Médicas y Nutrición Salvador Zubirán, Mexico City, Mexico.

Over 80% of children worldwide have been administered the bacillus Calmette-Guerin (BCG) vaccine for tuberculosis, with various immunological phenomena observed in relation to BCG.

**FIGURE 1:**
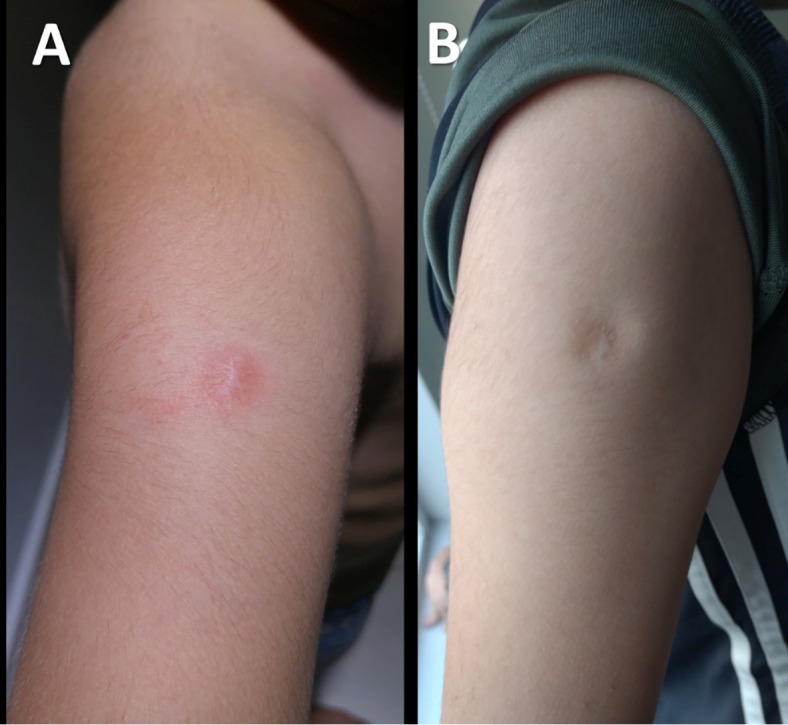
Panel A shows erythema at the site of the bacillus Calmette-Guerin (BCG) immunization scar two days after influenza vaccination. Panel B shows the same scar four months after vaccination with complete resolution of symptoms.

A nine-year-old Mexican boy presented with pain, swelling, and redness at the BCG scar (Figure 1A ) two days after an influenza vaccination (administered 3 cm from the BCG scar). The BCG scar was erythematous, indurated, and painful, while the influenza vaccination site remained unchanged. Vital signs were normal, with no signs of lymphadenopathy. The patient had a previous history of severe local reaction after BCG vaccination at 3 months of age (ulcer formation and nonsuppurative axillary lymphadenopathy). Additionally, the patient was tested positive for a tuberculin skin test at 3 years of age and received isoniazid/rifampin treatment. Erythema and induration at the BCG scar lasted 48 h, resolving without topical or systemic treatments (Figure 1B). 

BCG-vaccine interacts with epidermal macrophages, neutrophils, and dendritic cells, generating an intense immune response (reactive oxygen species, cytokines, and chemokines) and leading to cutaneous complications and scarring^1^. BCG scar inflammation has been described in patients with Kawasaki disease and other immune mediated events^1^ ^-^ ^2^. This is caused by cross-reactions between mycobacterial and human homologue heat shock proteins (HSP), specifically between mycobacterium HSP65 and human HSP63^3^. Elevated interleukin-1β (IL-1 β) and tumor necrosis factor-α (TNF- α) have been identified at the BCG scar in patients with Kawasaki disease^2^. In this case, a mechanism involving HSP liberation after influenza vaccination could have stimulated the immune response at the BCG scar^3^. 
